# Implementation of secondary reconstructions of flat-panel volume computed tomography (fpVCT) and otological planning software for anatomically based cochlear implantation

**DOI:** 10.1007/s00405-021-06924-0

**Published:** 2021-06-08

**Authors:** Franz-Tassilo Müller-Graff, Lukas Ilgen, Philipp Schendzielorz, Johannes Voelker, Johannes Taeger, Anja Kurz, Rudolf Hagen, Tilmann Neun, Kristen Rak

**Affiliations:** 1 Department of Oto-Rhino-Laryngology, Plastic, Aesthetic and Reconstructive Head and Neck Surgery and the Comprehensive Hearing Center, University of Wuerzburg, Josef-Schneider-Strasse 11, 97080 Wuerzburg, Germany; 2 Insitute for Diagnostic and Interventional Neuroradiology, University of Wuerzburg, Bavaria, Germany

**Keywords:** Cochlear duct length, Cochlear planning software, fpVCT, Secondary reconstruction, MSCT, Inter-electrode-distance

## Abstract

**Purpose:**

For further improvements in cochlear implantation, the measurement of the cochlear duct length (CDL) and the determination of the electrode contact position (ECP) are increasingly in the focus of clinical research. Usually, these items were investigated by multislice computed tomography (MSCT). The determination of ECP was only possible by research programs so far. Flat-panel volume computed tomography (fpVCT) and its secondary reconstructions (fpVCT_SECO_) allow for high spatial resolution for the visualization of the temporal bone structures. Using a newly developed surgical planning software that enables the evaluation of CDL and the determination of postoperative ECP, this study aimed to investigate the combination of fpVCT and otological planning software to improve the implementation of an anatomically based cochlear implantation.

**Methods:**

Cochlear measurements were performed utilizing surgical planning software in imaging data (MSCT, fpVCT and fpVCT_SECO_) of patients with and without implanted electrodes.

**Results:**

Measurement of the CDL by the use of an otological planning software was highly reliable using fpVCT_SECO_ with a lower variance between the respective measurements compared to MSCT. The determination of the inter-electrode-distance (IED) between the ECP was improved in fpVCT_SECO_ compared to MSCT.

**Conclusion:**

The combination of fpVCT_SECO_ and otological planning software permits a simplified and more reliable analysis of the cochlea in the pre- and postoperative setting. The combination of both systems will enable further progress in the development of an anatomically based cochlear implantation.

## Introduction

In cochlear implantation, best possible speech perception is desirable. Along with several other favourable prognostic factors in adults such as the motivation of the patient, medical history with occurrence of postlingual hearing loss and a short period of deafness, it is assumed that best speech perception can be reached by the correct electrode selection and an appropriate cochlear coverage [1–4]. Therefore, it is necessary to have sufficient data on the cochlear anatomy preoperatively, especially on the cochlear duct length (CDL). In recent years, new imaging technologies, models and formulas have been developed to measure the CDL [5–14]. Most﻿ of these studies were based on measurements of the CDL by Escudé in 2006 [5]. Most frequently, MSCT [5, 12] or cone-beam CT [8–10, 13, 14] have been used. In some studies, results were compared to more precise imaging, like micro-CT [9, 11, 13] or synchrotron radiation phase-contrast imaging [6, 7] as a reference method.

The flat-panel volume computed tomography (fpVCT) system has an innovative design which allows imaging of entire organs in one axial acquisition with an ultra-high spatial resolution [15]. Initial studies found superior image quality of the fine osseous temporal bone structures than in currently available MSCT scanners [16]. The first application of fpVCT in patients revealed a proper resolution regarding the critical structures for cochlear implantation [17]. Other studies first showed a significantly higher overall image quality compared to MSCT and a reduction of the effective dose of approximately 40% compared to 64-section- [18] and 128-section-MSCT [19]. Further advantages of fpVCT are portability-enabling the intraoperative use and reduced metallic artifacts, allowing postoperative position analyses, and more accurate frequency mapping of the electrode contacts [20–22]. As reported by Pearl et al. fpVCT offers the possibility to enhance image quality by secondary reconstructions (fpVCT_SECO_) [23], which can reach the same accuracy in measuring the CDL compared to experimental Micro-CT [24].

A specially designed software for otological surgical planning has been used, inter alia, to evaluate the possibility of facial nerve segmentation for otological training [25] and surgical planning of cochlear implantation in cases with post-meningitis ossification [26]. The clinical applicability of the software, in particular an angular insertion depth prediction for preoperative electrode selection has been demonstrated [27]. In various recently published studies, the CDL was also measured with this software, using MSCT and MRI [28–30]. They revealed low inter- as well as intraobserver variability and reliability [28, 29]. So far, evaluation of pre and postoperative clinical cochlear data created with fpVCT using the otological software has not been performed.

In the present study, different aspects should be investigated. Firstly, does the application of the fpVCT or fpVCT_SECO_ facilitate the preoperative measurements of the CDL in comparison to MSCT in the otological planning software, secondly, is it possible herewith to have a reliable measure of the postoperative CDL with fpVCT or fpVCT_SECO_ and thirdly, is there a difference in estimating the relative position of the electrode contacts in the comparison between fpVCT, fpVCT_SECO_ and MSCT?

## Methods

### Subjects and groups

In this retrospective single-center study, 30 patients from two cohorts were included, who were divided into three groups as depicted in Table 1.

**Table 1:** Data of the cohorts of patients used in this study

Cohort	Patients	Age	Implanted side		Group	Image modality	
						MSCT	fpVCT/ fpVCT_SECO_
I	20	64	12 Right	8 Left	1	Non-implanted	Non-implanted
					2	Non-implanted	Implanted
II	10	57	4 Right	6 Left	3	Implanted	Implanted

In one cohort, 20 patients were included who were implanted with a MED-EL FLEX^28^ electrode (28 mm electrode with 12 single contacts each 2.1 mm apart). All cochlear implantations were performed by experienced otosurgeons (KR, RH). For all patients, this was the first implanted ear. Mean age of the patients was 64 years (standard deviation (SD): 14.9 years). Surgery was performed for 12 patients on the right and for eight on the left ear. Every patient had a preoperative MSCT of the temporal bone and a postoperative fpVCT for position control of the implanted electrode. In addition, fpVCT_SECO_ was performed using the data of the fpVCT. Since radiological examinations of the patients include both temporal bones, different investigations with the three imaging modalities could be performed: in non-implanted ears (Group 1) and in preoperative non-implanted and postoperative implanted ears (Group 2).

Further images were evaluated in a second cohort of 10 patients, in which a MSCT and an fpVCT of an implanted ear was available. Consequently, the comparison of the three imaging modalities in implanted ears (Group 3) was possible. The mean age of this cohort was 57 years (SD: 21.2 years). Four subjects received their implant on the right side, six on the left side.

The retrospective anonymized study was conducted in concordance with local guidelines and principles of the Declaration of Helsinki and Good Clinical Practice and was approved by the local ethics committee at the University of Wuerzburg (2019020401).

### Imaging

FpVCT scans were performed using an angiographic unit (Axiom Artis; Siemens Healthcare AG, Erlangen, Germany) with commercially available software (Syngo DynaCT, Siemens).

The MSCT datasets were acquired using a SOMATOM Definition AS + (Siemens) with commercially available software (Syngo CT, Siemens). The standard application (inner ear high-resolution program) was applied using the following parameters: tube current = 38 mA; tube voltage = 120 kV; collimation = 0.6 mm; pitch = 0.55; slice thickness = 600 µm.

The fpVCT datasets were acquired using the following parameters: 20 s DCT Head protocol; tube current = 21 mA; tube voltage = 109 kV; rotation angle = 200 degree; pulse length = 3.5 ms; frame angulation step = 0.5 degree/frame; slice thickness = 466 µm.

**Fig. 1:**
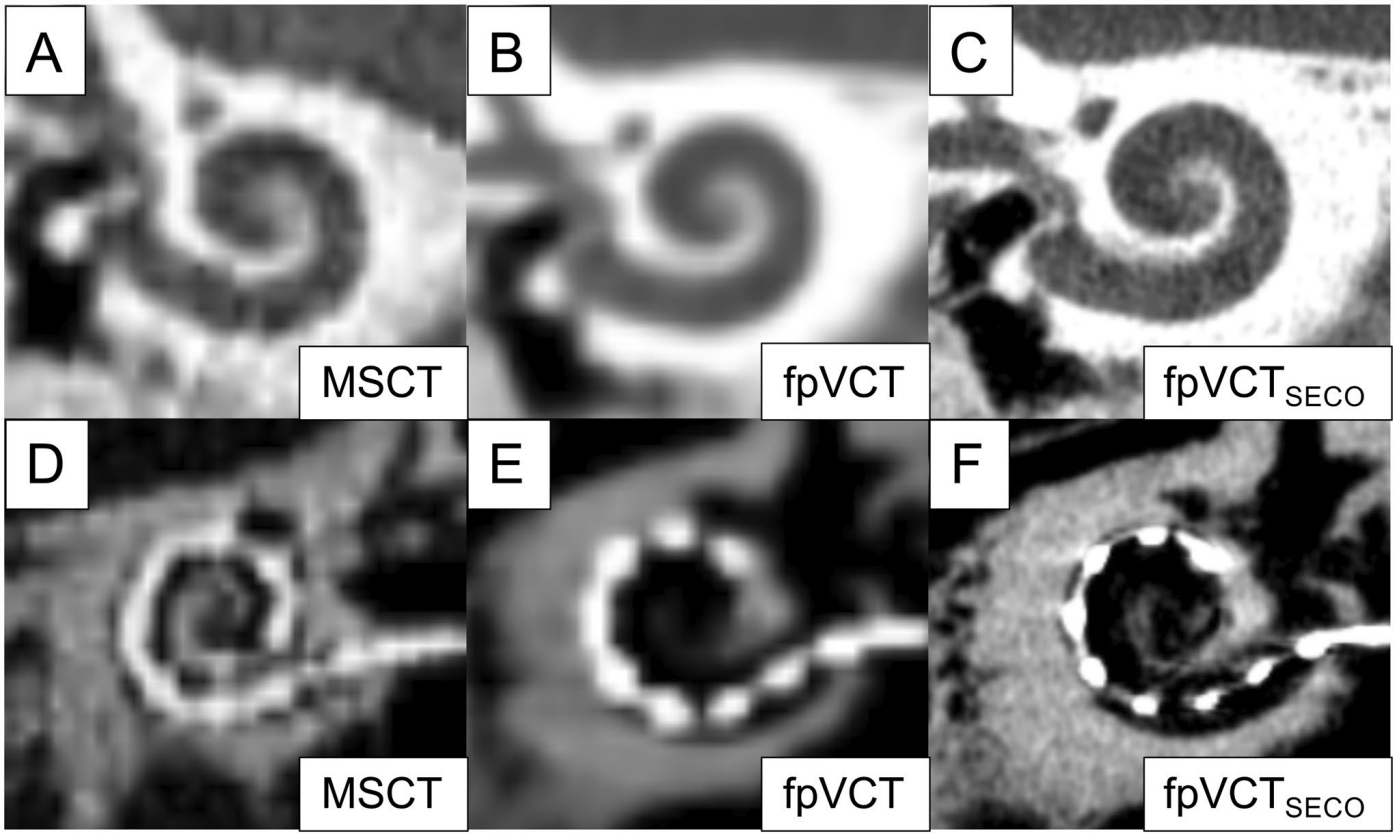
“Cochlear view” of different imaging modalities and settings. Representative image of MSCT (600 µm) is shown in panel (**A**), display of fpVCT (466 µm) in panel (**B**). Image of fpVCT_SECO_ (99 µm) is presented in panel (**C**). Respectively, panels (**D**–**F**) demonstrate the “Cochlear View” [31] with an implanted electrode. MSCT: multislice computed tomography, fpVCT: flat-panel volume computer-tomography, fpVCT_SECO_: fpVCT secondary reconstruction

From these datasets, fpVCT_SECO_ were designed according to the findings of Pearl et al. [23] using the following settings: 512 × 512 section matrix; HU kernel types; sharp image characteristics; slice thickness = 99 µm. Visual comparison of different imaging modalities and settings is presented in Fig. 1A–F.

### Measurement of cochlear parameters and calculating CDL_OC_

**Fig. 2:**
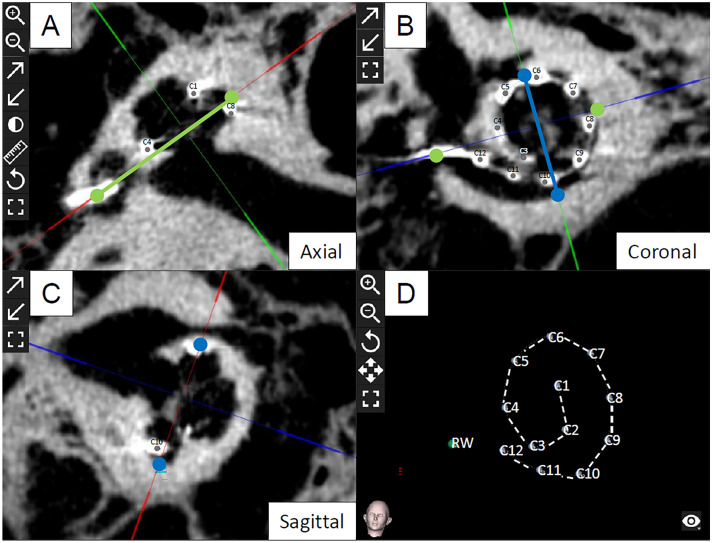
Evaluation of cochlear parameters. Visualization of the cochlear parameters and the electrode contacts in the different body axes using fpVCT_SECO_ in the otological planning software. The “axial view” is shown in panel (**A**), the “coronal”, known as the “Cochlear View” [32] in panel (**B**) and the “sagittal view” in panel (**C**). Three-dimensional display of the inserted implant electrodes after identifying them in the three-body axes is shown in panel (**D**). Diameter A is shown as green fiducials and line, width B, respectively, as blue fiducials and line. From these data, CDL_OC_ was calculated. C1-12: Electrode contacts. *RW* Round window

To determine pre- and postoperative cochlear parameters, OTOPLAN^®^ software (CAScination AG (Bern, Switzerland) in cooperation with MED-EL (Innsbruck, Austria), version 2) was used. The data sets were converted to the DICOM standard by the PACS network of the hospital and transferred anonymously to the software using a memory stick. In all images initially, the coronal oblique view, typically referred to as “Cochlear View” [31, 32], was created by 3-dimensionally rotating against the axial, sagittal and coronal axis (Fig. 1). After attempting to receive the most optimized view, the cochlear diameter (A) and width (B) were measured. Both, the process of orientation and landmark selection, were carried out in two test series with an interval of two weeks by one medical examiner, who is trained as an ENT-specialist and is very experienced in the evaluation of cochlear imaging. Patient’s data were anonymized and the order of the evaluation of both the imaging modality as well as the temporal bones was randomized. The cochlear A-value is defined as a straight line from the round window, passing the modiolus, to the furthest point on the opposite wall of the cochlea. The cochlear B-value is the straight line connecting the two opposite lateral walls of the cochlea, perpendicular to the cochlear diameter passing through the modiolus. Visualization of the parameters in the different body axes is shown in Fig. 2A–C. From these data, the CDL of the organ of Corti (CDL_OC_) was calculated from the otological software using Eq. (1), which has been originally described as the “elliptic circular approximation” [9]. Since the OC does not start at the center of the round window but reaches into the cochlear base, the equation in the software version used herein has been supplemented by the length of this so-called hook region of 1.58 mm as follows:

1 $${\text{CDL}}_{{{\text{OC}}}} \; = \;\left( {{1}.{71}\; \times \;\left( {{1}.{18}\; \times \;\left( {A_{{{\text{OC}}}} } \right)\; + \;{2}.{69}\; \times \;B_{{{\text{OC}}}} } \right)\; - \;\sqrt {\left( {0.72\; \times \;A_{{{\text{OC}}}} \; \times \;B_{{{\text{OC}}}} } \right)} \; \times \;0.{18}} \right)\; + \;{1}.{58}$$

### Determination of electrode contact position

The electrode contact position (ECP) of the electrodes was determined to perform postoperative position control. Since all patients received a device with 12 electrodes, 12 fiducials were, respectively, placed by rotating through the 3D-illustrated “Cochlear View” [31]. The fiducials were placed as precisely as possible at the center of each electrode contact of the three body axes (Fig. 2A–C). From these data the otological planning software calculated the insertion depth in mm for every single electrode. For statistical analysis, the distances between every single electrode (inter-electrode distance, IED) were extracted. The entire electrode array can be seen after these maneuvers and rotated in every direction in a three-dimensional display, as seen in Fig. 2D.

### Statistics

Before parametrical analyses were conducted, the normal distribution of all data series was confirmed by Kolmogorov–Smirnov and Shapiro–Wilk test. As a requirement for paired *t*-test, normal distribution of the differences between both paired groups was also checked. The paired *t*-test was used for reference comparison between the two measurement series of the particular imaging modalities. Differences with a p-value of less than 0.05 were considered to be statistically significant. For evaluation of the differences of the absolute mean values of the CDL_OC_ in the three modalities and settings, one-way repeated measures analysis of variance (ANOVA) was applied. When the data were not normally distributed, the Friedman-Test was used.

Intraclass correlation (ICC) was performed for evaluating intraobserver variability in each modality and setting within all test series. Based on the publication of Wirtz et al., a two-way mixed model was performed, and single measure values of ICC were used [33]. ICC was tested for absolute agreement. As described by Cicchetti et al., ICC values were interpreted by applying the following scale: unacceptable (ICC < 0.4), fair (0.4 ≤ ICC < 0.6), good (0.6 ≤ ICC < 0.75) and excellent (0.75 ≤ ICC) [34]. The associated Cronbach’s alpha (CA) was calculated according to the following scale: unacceptable (*α* < 0.7), fair (0.7 ≤ *α* < 0.8), good (0.8 ≤ *α* < 0.9) and excellent (0.9 ≤ *α*) [34]. For ICC and CA only significant results were considered.

According to Koch et al., a clinically acceptable margin of divergence of ± 1.5 mm for CDL_OC_ was assumed [7]. With a recalculation from the same study, the clinically acceptable variance of ± 0.09 mm for IED was derived.

Statistical analyses and creating diagrams were performed by GraphPad Prism (Version 8.4.0, San Diego, California, USA), as well as IBM SPSS Statistics (Version 25.0.0.0; IBM Corporation, Armonk, New York USA). Data are presented in bar charts and Bland–Altman-Plots.

## Results

### Population parameters

All parameters of the measurements carried out, including mean values, ranges, SD, 95%-confidence intervals (CI), ICC, CA and *t*-Test for both cohorts, are depicted in Table 2.

**Table 2:** Analysis of non-implanted and implanted ears

	Non-implanted ears	Preoperative non-implanted and postoperative implanted ears	Implanted ears	Implanted ears
Group	1	2	3	3
Patients	20	20	10	10
Number of test series	2	2	2	2

Test series	CDL_OC_ (mm)	CDL_OC_ (mm)	CDL_OC_ (mm)	IED (mm)
MSCT postoperative (600 µm)				
ICC	0.807^a^	0.862^a^	0.785^a^	0.644^b^
95%-CI	0.324^d^–0.935	0.578^c^–0.950	0.336^d^–0.942	0.077^d^–0.898
Cronbach’s alpha	0.934^a^	0.946^a^	0.869^b^	0.776^c^
* t*-Test	0.0011*	0.0077*	0.7499	0.4706
Mean	34.55	34.29	37.59	2.55
Range	31.20–36.95	30.50–37.15	34.35–42.40	2.20–2.81
SD	1.60	1.81	2.12	0.16
95%-CI	1.50	1.70	3.05	0.24
fpVCT postoperative (466 µm)				
ICC	0.768^a^	0.946^a^	0.942^a^	0.875^a^
95%-CI	0.465^c^–0.905	0.578^c^–0.959	0.783^a^–0.985	0.568^c^–0.968
Cronbach’s alpha	0.890^b^	0.946^a^	0.967^a^	0.926^a^
*t*-Test	0.0297*	0.1211	0.9347	0.9646
Mean	34.63	36.43	37.01	2.51
Range	31.55–37.60	33.55–39.30	34.70–42.00	0.12
SD	1.47	1.67	2.06	2.23–2.67
95%-CI	1.38	1.57	2.95	0.19
fpVCT_SECO_ postoperative (99 µm)				
ICC	0.926^a^	0.904^a^	0.961^a^	0.936^a^
95%-CI	0.823^a^–0.970	0.772^a^–0.961	0.854^a^–0.990	0.765^a^–0.984
Cronbach’s alpha	0.960^a^	0.947^a^	0.978^a^	0.964^a^
* t*-Test	0.6359	0.9782	0.6529	0.8220
Mean	35.84	36.18	36.55	2.50
Range	32.95–38.45	32.90–39.60	34.55–41.40	2.22–2.68
SD	1.39	1.75	2.07	0.14
95%-CI	1.30	1.64	2.98	0.21

Overall comparison of intraobserver variability and statistical data of CDL_OC_ measurements of each imaging modality and setting

^a^Excellent—ICC:﻿ Cronbach’s alpha

^b^Good—unacceptable 0.000–0.400: unacceptable 0.000–0.700

^c^Fair—fair 0.400–0.600: fair 0.700–0.800

^d^Unacceptable—good 0.600–0.750: good 0.800–0.900

*Significant—excellent 0.750–1.000: excellent 0.900–1.000

#### Group 1: determination of CDL_OC_ in non-implanted ears by MSCT, fpVCT and fpVCT_SECO_

**Fig. 3:**
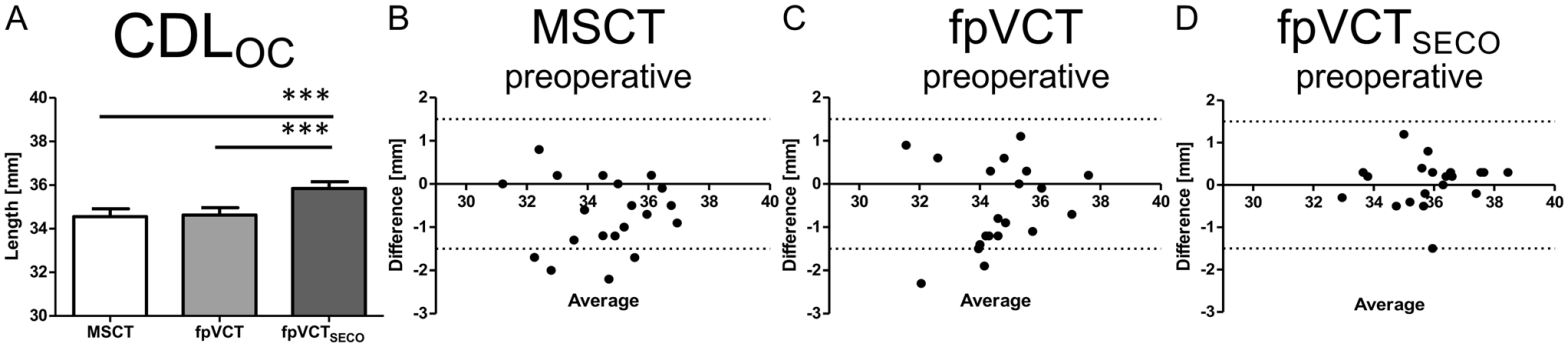
Analysis of non-implanted ears with comparison of different radiological settings and modalities in a clinical setting (Group 1). Bar shows absolute values of measurements of the CDL_OC_ based on MSCT, fpVCT and fpVCT_SECO_ (**A**). Bland–Altman plots were constructed to visualize discrepancies within the two test series of the various imaging settings and modalities (**B**–**D**). According to Koch et al. [7], a clinically acceptable deviation of ± 1.5 mm was assumed analyzing CDL_OC_. Differences between the cohorts are indicated as significant, ***p ≤ 0.001. MSCT: multislice computed tomography, fpVCT: flat-panel volume computed tomography, fpVCT_SECO_: fpVCT secondary reconstruction

CDL_OC_ were measured in non-implanted ears. The mean value for the length of the CDL_OC_ were 34.55 mm (range: 31.20–6.95 mm, SD: 1.60 mm) measured with MSCT. For fpVCT, it was 34.63 mm (range: 31.55–37.60 mm, SD: 1.47 mm) and for fpVCT_SECO_, 35.84 mm (range: 32.95–38.45, SD: 1.36 mm) were determined (Fig. 3A). All values measured by fpVCT_SECO_ differed significantly from those evaluated using MSCT and fpVCT (*p* < 0.001). There were no significant differences in the measurements between MSCT and fpVCT.

When examining CDL_OC_, there was a significant difference within the two test series using MSCT (*p* = 0.0011) and fpVCT (*p* = 0.0297), but not applying fpVCT_SECO_. ICC were consistently categorized as excellent for all imaging modalities. The lower limits of the CI were unacceptable (MSCT), fair (fpVCT), and excellent (fpVCT_SECO_). CA was classified as excellent for MSCT and fpVCT_SECO_, and as good for fpVCT (Table 2).

Considering corresponding Bland–Altman-Plots for CDL_OC_, which demonstrate clinically acceptable values, MSCT had four errors (Fig. 3B). Measurements with fpVCT showed two deviations (Fig. 3C) and assessing fpVCT_SECO_, there were no divergencies (Fig. 3D).

Overall, fpVCT_SECO_ was categorized as excellent and had no clinically unacceptable deviations when measuring cochlear parameters in cochleae without an inserted electrode.

#### Group 2: measurements of CDL_OC_ by using non-implanted preoperative MSCT and postoperative fpVCT and fpVCT_SECO_ with an inserted electrode

**Fig. 4:**
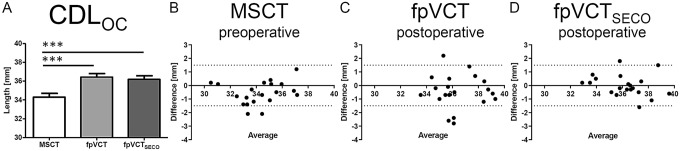
Analysis of preoperative non-implanted and postoperative implanted ears (Group 2). Bar shows absolute mean values of measurements of CDL_OC_ based on MSCT, fpVCT and fpVCT_SECO_ (**A**). Bland–Altman plots were constructed to visualize discrepancies within the two test series of the various imaging settings and modalities (**B**–**D**). According to Koch et al. [7], a clinically acceptable deviation of ± 1.5 mm was assumed analyzing CDL_OC_. Notably, MSCT-measurements were done preoperatively without inserted electrode and fpVCT/ fpVCT_SECO_ measurements postoperatively. Differences between the different cohorts are indicated as significant, ***p ≤ 0.001. MSCT: multislice computed tomography, fpVCT: flat-panel volume computed tomography, fpVCT_SECO_: fpVCT secondary reconstruction

To evaluate whether it is possible to have a reliable postoperative measurement with fpVCT or fpVCT_SECO_ despite the inserted electrode, CDL_OC_ were measured pre- and postoperatively. The mean values for the length of the CDL_OC_ were 34.29 mm (range: 30.50–37.15 mm, SD: 1.81 mm) measured with preoperative MSCT. For postoperative fpVCT, it was 36.43 mm (range: 33.55–39.30 mm, SD: 1.67 mm) and for postoperative fpVCT_SECO_ 36.18 mm (range: 32.90–39.60, SD: 1.75 mm) (Fig. 4A). The values measured in MSCT differed significantly from those evaluated with fpVCT and fpVCT_SECO_ (*p* < 0.001 for CDL_OC_). Between fpVCT and fpVCT_SECO_ measurements, no significant differences were observed.

Regarding CDL_OC_, there was a significant difference within the test series for MSCT (*p* = 0.0077), but not for fpVCT and fpVCT_SECO_. ICC and CA were consistently rated to be excellent. The lower limit of the CI was only excellent for fpVCT_SECO_ and fair for MSCT and fpVCT (Table 2). Summarising, using fpVCT_SECO_ to evaluate CDL_OC_ postoperatively was exclusively excellent.

In the corresponding Bland–Altman-Plots, using MSCT produced two clinically unacceptable deviations for CDL_OC_ (Fig. 4B). Measurements with fpVCT showed four aberrations for and (Fig. 4C) assessing fpVCT_SECO_, there were two outlier (Fig. 4D).

#### Group 3: comparison of CDL_OC_ in cochleae with an inserted electrode and evaluation of the IED by using MSCT, fpVCT and fpVCT_SECO_

**Fig. 5:**
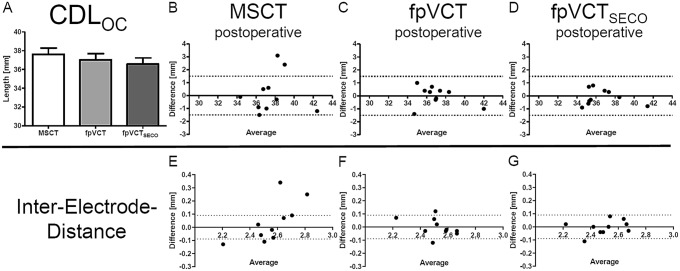
Analysis of implanted ears (Group 3). Bars shows absolute values of measurements of CDL_OC_ based on MSCT, fpVCT and fpVCT_SECO_ (**A**). Bland–Altman plots were constructed to visualize discrepancies within the two test series of the various imaging settings and modalities (**B**–**D**). According to Koch et al. a clinically acceptable error of ± 1.5 mm was assumed analysing CDL_OC_. Moreover, Bland–Altman plots of MSCT (**E**), fpVCT (**F**) and fpVCT_SECO_ (**G**) were constructed to demonstrate variations within the two test series localizing the IED in postoperative analysed ears. Derived from Koch et al. a clinically acceptable error was calculated with ± 0.09 mm for IED. MSCT: multi-slice computed tomography, fpVCT: flat-panel volume computer-tomography, fpVCT_SECO_: fpVCT secondary reconstruction

This investigation was performed in cases with an MSCT and fpVCT scan with an inserted electrode. The mean value for CDL_OC_ was 37.59 mm (range: 34.35–42.40 mm, SD: 2.12 mm) using MSCT. Assessing fpVCT, it was 37.01 mm (range: 34.70–42.00 mm, SD: 2.06 mm). Measurements with fpVCT_SECO_ revealed a CDL_OC_ of 36.55 mm (range: 34.55–41.40 mm, SD: 2.07 mm) (Fig. 5A). All values demonstrated above did not differ significantly between the different imaging modalities.

For CDL_OC_, no significant differences were found within the two test series and ICC was consistently ranked as excellent for all imaging modalities. CA was stated excellent for fpVCT and fpVCT_SECO_ but just good for MSCT. The lower limit of the CI was unacceptable for MSCT, while it was excellent for fpVCT and fpVCT_SECO_ (Table 2).

To demonstrate a clinically arguable zone, corresponding Bland–Altman-Plots for CDL_OC_ were created. For MSCT, 2 clinically unacceptable deviations became apparent (Fig. 5B). For fpVCT, no deviances were observed (Fig. 5C) and using fpVCT_SECO_ no aberrations were measured (Fig. 5D).

Furthermore, for the purpose of evaluating the accuracy of the determination of electrode contact positions in the different imaging modalities, distances between every single electrode (IED) of the implant were extracted from the otological planning software. A statistical workup of the mean was not performed since different electrodes in different patients have been used. Therefore, a comparison of the three imaging modalities by ICC was carried out. No significant difference was found within the two test series. ICC was good and CA fair for MSCT. In contrast, the same parameters were excellent when using fpVCT and fpVCT_SECO_ (Table 2). The corresponding Bland–Altman-Plots demonstrate four clinically unacceptable deviations for MSCT, two clinically unacceptable aberrations for fpVCT and one clinically unacceptable value for fpVCT_SECO_ (Fig. 5E–G).

## Discussion

In the field of cochlear implantation there is a growing interest in determining the cochlear anatomy and the intracochlear position of an implanted electrode, as it is assumed that an anatomically optimal location of the electrode can lead to a better hearing outcome after surgery [35]. For the preoperative planning and the postoperative position control, new high-resolution imaging techniques as well as newly developed otological planning softwares can be used. In order to evaluate whether fpVCT and its secondary reconstruction (fpVCT_SECO_) contributes to anatomically based cochlear implantation compared to the conventionally used MSCT, data of the different imaging modalities were analyzed using a newly developed otological planning software in a clinical setting.

The three different imaging modalities were compared measuring the clinically most important parameter CDL_OC_. The comparison of MSCT and fpVCT using raw data stacks with similar slice thicknesses revealed only moderate clinical discrepancies. In contrast, clinical relevant differences were seen, when compared to fpVCT_SECO_ with low slice thickness, which was the only one that was categorized as excellent in all clinical settings, including postoperative scans with an implanted electrode. Moreover, even the lower limits of the 95%-CI were consistently ranked as excellent. In comparison to MSCT, where unacceptable deviations in non-implanted ears occurred (20% in Group 1 (Fig. 3B) and 10% in Group 2 (Fig. 4B)), there were no clinically unacceptable deviations measuring CDL_OC_-values using fpVCT_SECO_ (Fig. 3D). Moreover, in implanted ears (Group 3), there were no divergencies using fpVCT_SECO_ (Fig. 5D) compared to deviations in 20% of the cases using MSCT (Fig. 5B). These results indicate low intra-variability for fpVCT_SECO_ measuring CDL_OC_ and thus good clinical applicability. This is in accordance with Rathgeb et al., who was among the first to evaluate a good clinical observer variability of the planning software [27]. FpVCT without secondary reconstruction is considered a precise method for assessing the parameters of the inner ear with the same or even less radiation dose than conventional methods [18, 19]. This might reduce the risk of radiogenic damage in the long term [36, 37] while increasing diagnostic image quality [18, 19, 22]. The option of generating secondary reconstructions limits the need for further radiation exposure, while the image quality is even more greatly improved. Indeed, the use of fpVCT_SECO_ in this study, revealed the above mentioned advantages, in addition to the obvious reduced metal artifacts [20, 21]. The demonstrated results are in accordance with recently published data on measurements of CDL_OC_ using fpVCT_SECO_ in temporal bone specimen [24] using multi-planar reconstructions [38]. This might be well explained by the higher resolution of fpVCT_SECO_ with a slice thickness of 99 µm in comparison to the other image qualities. Summarized, the findings of this study implicate that the application of fpVCT_SECO_ facilitates the measurement of the CDL_OC_ in clinical imaging and makes them more reproducible in comparison to MSCT using this otological planning software.

The mean values of all measurements, depicted in Fig. 3A and Fig. 4A (regardless with or without implanted electrode) for the CDL_OC_ using fpVCT_SECO,_ were 36.14 mm and differed significantly between measurements with MSCT (34.55 mm) and fpVCT (35.66 mm). That is in contrast to studies on measurements of CDL_OC_ using the corresponding software. The authors described a shorter mean length for CDL_OC_ with 32.91 mm [39] and 32.84 mm [29]. This may be explained by the fact that only conventional MSCT was used. In addition, as the exact version of the software was not mentioned in these publications, it might be possible that older versions have been used that did not include the hook region yet in the equation as it was in Version 2 of the software in this study. Moreover, it was suggested that any effect of slice thickness on the CDL measurement is likely rather small. However, it could not be ruled out the possibility that variation in other parameters affecting CT quality could result in greater variability in CDL estimates as this present study has now confirmed [29]. A systematic error was described in recent studies when the points at the lateral wall are placed more medially. This occurs mainly in scans with lower resolution than in high-resolution scans of micro-CT or pictures of histological sections [9, 10]. This induces shorter values of cochlear parameters. This phenomenon might interestingly also explain why the values measured by MSCT with implanted electrodes, as depicted in Fig. 5A–C, are not even more significantly different and even slightly higher than those measured with fpVCT_SECO_. Due to the larger artifacts of the electrodes in MSCT, the points marking the lateral wall might be set more laterally than in the true anatomical set, responsible for the discrepancies in different parameters. Therefore, clinical analyses using fpVCT_SECO_ probably represents the true length, since assessments with micro-fpVCT and micro-CT in temporal bone specimens have shown no significant different CDL at the lateral wall values up to over 40 mm [13, 24], which corresponds to a CDL_OC_ of about 35 mm. In line with this, in other studies, shorter CDL values have been reported in clinical modalities [8, 13]. Based on these and the presented findings, it should be carefully considered whether it might be better to use longer electrodes than currently assumed for covering the correct CDL_OC_, when measured by MSCT or other low-resolution images. This might also result in even more accurate predictions for preoperative angular insertion depths, some of which have already been calculated with the planning software [27].

In order to verify the postoperative location of the implant, IED was computed from the data generated in the otological planning software. It was analyzed which imaging modality had the highest accuracy to detect the IED of 2.1 mm of the FLEX^28^-electrode. Major differences between MSCT and fpVCT_SECO_ were observed. Regarding the reproducibility, MSCT was categorized as good, whereas fpVCT_SECO_ as excellent. The differences become even clearer, when focusing on the lower limit of the 95%-CI interval, where MSCT was unacceptable, compared to excellent for fpVCT_SECO_. Moreover, the presented study revealed that MSCT showed clinically unacceptable deviations in 40% of the cases (4/10) (Fig. 5E), while using fpVCT_SECO_, there was only one clinically unacceptable value (10%) (Fig. 5G). To the best of our knowledge, up to date, this is the first study presenting the IED measured in the used otological planning software. However, in several studies, it was shown that fpVCT is very appropriate for postoperative examination following cochlear implantation, in particular, to determine the final position of the electrode arrays [22, 23, 40], but in none of these publications the intra-electrode variables were measured. The increased clinically unacceptable errors using MSCT in this study are presumably due to radiological artifacts. Indeed, it has already been shown that the real diameter of the electrode is half in comparison to the radiological one in CBCT [41]. Even rather, a study reported that single electrode contacts are only visible in fpVCT and not in MSCT [22]. This may be of clinical relevance, as it is assumed that a regular tonotopic stimulation of the cochlea by the electrode could lead to improved hearing performance and a better speech perception [42–44]. Therefore, the wide variation of the measurements using MSCT could lead to an incorrect calculation of frequency mapping. Jiam et al. demonstrated that based on fpVCT_SECO_ imaging findings, 83% of the electrode contacts, in which standardized frequency maps were used, might be improved by reprogramming and concluded that individual pitch mapping should be performed [35]. These pitch maps would be even more accurate if the particular electrode position was located as precisely as possible.

It needs to be mentioned that only one electrode type from one implant-firm was used for measuring IED. This is due to the use of the otological planning software, which only allows the use of the manufacture’s electrodes. However, it is known that certain electrodes are more prone to generate artefacts, which are believed to depend on the thickness and material of the metal [45]. Therefore, we cannot exclude that the problems of MSCT measuring the IED may be more a problem of the electrode rather than of the modality.

One potential limitation of this study is the sample size concerning pre- and postoperative measurements. In addition, the study has the limitation to observe only intraobserver variability, but low interobserver variability in the use of this otologic planning software has been shown before [27–29]. We also cannot rule out a possible influence of the investigator’s training curve. However, since there was a blinded and randomized sequence not only of the patient data but also of the imaging modalities, such an effect would affect all modalities.

## Conclusion

The results of the presented study suggest that the combination of fpVCT_SECO_ and otological planning software will enable further progress in the development of an anatomically based cochlear implantation. It might be beneficial to perform the preoperative planning on cochlear implantation regarding the correct electrode selection with fpVCT_SECO_ utilizing the otological planning software. Furthermore, postoperative control of the IED with fpVCT_SECO_ will enhance accuracy of creating individual pitch maps through the software. This might further improve the exact determination of cochlear anatomy and refine postoperative frequency mapping for a better hearing outcome.

## Data Availability

The data of the measurements generated in this study are available upon reasonable request from the corresponding author.
